# Emotional processes and stress in children affected by hereditary angioedema with C1-inhibitor deficiency: a multicenter, prospective study

**DOI:** 10.1186/s13023-018-0871-x

**Published:** 2018-07-13

**Authors:** Livia Savarese, Maria Bova, Raffaella De Falco, Maria Domenica Guarino, Raffaele De Luca Picione, Angelica Petraroli, Riccardo Senter, Claudia Traverso, Matteo Zabotto, Andrea Zanichelli, Eugenio Zito, Maria Alessio, Mauro Cancian, Marco Cicardi, Adriana Franzese, Roberto Perricone, Gianni Marone, Paolo Valerio, Maria Francesca Freda

**Affiliations:** 1 Department of Humanities, University Federico II, via Porta di Massa 1, 80133 Naples, Italy; 2 Department of Translational Medical Sciences and Center for Basic and Clinical Immunology Research (CISI), University of Naples Federico II, Naples, Italy; 3 Department of Clinical Neuroscience, Anesthesiology and Drug Administration, University Federico II, Naples, Italy; 4 Rheumatology, Allergology and Clinical Immunology, Department of “Medicina dei Sistemi”, University of Rome Tor Vergata, Rome, Italy; 5 Department of Medicine, University of Padua, Padua, Italy; 6 Department of Pediatrics, Rheumatology Unit, University Federico II, Naples, Italy; 7 Department of Psychiatry, “Luigi Sacco” Hospital, University of Milan, Milan, Italy; 8 ASST Fatebenefratelli Sacco, Department of Biomedical and Clinical Sciences, “Luigi Sacco” Hospital, University of Milan, Milan, Italy; 9 Department of Social Sciences, University Federico II, Naples, Italy

**Keywords:** Alexithymia, C1-inhibitor deficiency, Children, Hereditary angioedema, Psychological factor, Stress

## Abstract

**Background:**

Hereditary angioedema with C1-inhibitor deficiency (C1-INH-HAE) is characterized by recurrent edema of unpredictable frequency and severity. Stress, anxiety, and low mood are among the triggering factors most frequently reported. Impaired regulation and processing of emotions, also known as alexithymia, may influence outcomes. The aim of this study was to confirm the presence of alexithymia and stress in children with C1-INH-HAE, to determine whether they are also present in children affected by other chronic diseases, and to investigate their relationship with C1-INH-HAE severity. Data from children with C1-INH-HAE (*n* = 28) from four reference centers in Italy were compared with data from children with type 1 diabetes (T1D; *n* = 23) and rheumatoid arthritis (RA; *n* = 25). Alexithymia was assessed using the Alexithymia Questionnaire for Children scale; perceived stress was assessed using the Coddington Life Event Scale for Children (CLES-C).

**Results:**

Mean age (standard deviation [SD]) in the C1-INH-HAE, T1D, and RA groups was 11.8 (3.3), 11.7 (2.9), and 11.1 (2.6) years, respectively. Mean C1-INH-HAE severity score was 5.9 (2.1), indicating moderate disease. Alexithymia scores were similar among disease groups and suggestive of difficulties in identifying and describing emotions; CLES-C scores tended to be worse in C1-INH-HAE children. C1-INH-HAE severity was found to correlate significantly and positively with alexithymia (*p* = 0.046), but not with perceived stress. Alexithymia correlated positively with perceived stress.

**Conclusions:**

Alexithymia is common in children with chronic diseases. In C1-INH-HAE, it may result in increased perceived stress and act as a trigger of edema attacks. Comprehensive management of C1-INH-HAE children should consider psychological factors.

**Electronic supplementary material:**

The online version of this article (10.1186/s13023-018-0871-x) contains supplementary material, which is available to authorized users.

## Background

Hereditary angioedema with C1-inhibitor deficiency (C1-INH-HAE) is a rare autosomal dominant disorder with a reported prevalence of approximately 1 case per 50,000 persons [1, 2]. It is caused by mutations in the *SERPING1* gene encoding the serpin-type protease inhibitor C1-INH [1]. Quantitative or qualitative deficiency of C1-INH results in the uncontrolled activation of the complement and contact pathways leading to the release of vasoactive substances including bradykinin, a key mediator of enhanced vascular permeability and edema formation [2]. C1-INH-HAE is characterized by recurrent subcutaneous and/or submucosal edema, without wheals or pruritus. Clinical manifestations exhibit high intra- and inter-individual variability and angioedema attacks occur with unpredictable frequency and severity. Swelling attacks typically affect the skin, upper airway, and gastrointestinal tract; abdominal attacks can be very painful and laryngeal attacks can be life-threatening due to the risk of asphyxiation [2]. C1-INH-HAE imposes a considerable burden on affected patients and their families, during and between angioedema episodes [3–6]. The reported age of onset of C1-INH-HAE attacks ranges from 4 to 18 years [7]. In children, C1-INH-HAE significantly impairs quality of life (QoL) and is associated with increased anxiety levels [8, 9].

The reasons for the high variability and unpredictability of C1-INH-HAE manifestations are still poorly understood [10]. Although most attacks appear to occur spontaneously, some factors triggering the onset of angioedema are known, including emotional stress and anxiety, physical trauma, medical or dental procedures, infection, and changes in estrogen levels [11, 12]. Emotional states are known to influence physical health [13]. Indeed, stress, anxiety, and low mood are the most frequent triggers reported by patients with C1-INH-HAE [14]. At the same time, C1-INH-HAE itself causes anxiety and depression, which leads to a vicious circle of increased emotional stress and higher attack frequency [14, 15]. In recent years, several studies have suggested the interaction of the immune system with the central nervous system to explain the effects of mood and anxiety on inflammatory diseases [15, 16]. A study in patients with C1-INH-HAE showing an increase in sympathetic activation at rest and a blunted response to experimentally induced stress have suggested that autonomous nervous system function may be altered [17]. Overall, studies addressing specifically the role of stress and psychological factors in C1-INH-HAE are still very limited, especially in children [10, 18, 19].

In a recent study we evaluated, for the first time in children, the psychological profile, the perceived level of stress, and the ability to cope with emotions of 12 children with C1-INH-HAE [18]. The majority of parents, who were also recruited in the study, indicated emotional stress as a trigger of angioedema attacks. Stress levels measured using the Coddington Life Events Scale (CLES) appeared to be higher in children with C1-INH-HAE compared with age-matched healthy controls. Furthermore, all children showed impaired ability to recognize and describe their feelings, as assessed by the Alexithymia Questionnaire for Children (AQC) and the Level of Emotional Awareness Scale for Children. The concept of alexithymia refers to the lack of words for emotions and was introduced in the early 1970s from the observation of patients with classic psychosomatic diseases [20–22]. The concept encompasses various deficits in the cognitive processing of emotions, including difficulty in identifying feelings, difficulty in describing feelings to others, externally oriented thinking, and a limited imaginative capacity [22]. Alexithymia is considered a key element in the psychosomatic process and in the neurologic mechanisms causing physical illness [23]. The presence of alexithymia is generally considered to be an unfavorable characteristic for disease control and health promotion [24].

We present here the results of a study that we conducted to further evaluate the relationship between emotional processes and disease manifestations in children with C1-INH-HAE and in two control groups, namely children with type 1 diabetes (T1D) and rheumatoid arthritis (RA). The objectives of the study were: 1) to confirm the presence of alexithymia and high levels of perceived stress in a larger population of C1-INH-HAE children; 2) to determine whether the presence of alexithymia and stress is specific for C1-INH-HAE; and 3) to establish whether C1-INH-HAE severity correlates with alexithymia, perceived stress and anxiety.

## Methods

### Study design and patients

This was a multicenter, exploratory study conducted in children with C1-INH-HAE to investigate the presence of psychological factors and their correlation with disease severity. The study also included two control populations consisting of children affected by other chronic diseases, namely T1D and RA. T1D patients were selected as control because T1D is the chronic condition with the highest incidence among children and has well-established protocols for disease control and patient management in the pediatric setting. RA patients were selected as this condition is characterized by a high variability of symptoms in children similar to C1-INH-HAE.

Patients and their parents were recruited via phone calls, based on medical records and with cooperation of their physicians. C1-INH-HAE patients were recruited from the center coordinating the study (University Hospital Federico II, Naples, Italy) and from three additional HAE reference centers in Italy (Rome, Padua, and Milan). T1D and RA patients were recruited from the departments of pediatric diabetes and pediatric rheumatology of the coordinating center. Patients were aged ≥6 years and ≤ 17 years and had been diagnosed with their chronic condition for at least 2 years. All procedures performed in this study were in accordance with the ethical standards of the study coordinating center and with the 1964 Helsinki Declaration and its later amendments or comparable ethical standards. Prior to study participation, patients and their parents signed an informed consent. Children received age-specific versions of the informed consent document [18].

### Assessments

Demographic and clinical data were collected from patient medical charts and diaries. Study assessments were performed during two face-to-face meetings for each study patient; the first meeting (45 min) was with the parents and the second meeting was with the child (90 min, approximately). At the first meeting, parents were administered the questionnaire Child Behavior Check-List, based on which the presence of psychopathological conditions was ruled out [25, 26]. During the meeting with the child, perceived stress and psychological factors were evaluated using the following tools: Coddington Life Events Scale for Children (CLES-C); Alexithymia Questionnaire for Children (AQC), or Toronto Alexithymia Scale 20 (TAS-20) in children aged > 15 years; Physiological Hyperarousal in Children (PH-C). The questionnaires were administered to the children by trained personnel; validated Italian translations of the questionnaires were used. The CLES-C is a validated and well-established 36-item questionnaire, which measures the frequency and timing of both positive and negative life events relevant for this age group during the last year (four trimesters) [27, 28]. By measuring significant life events in terms of Life Change Units, the CLES-C can provide insight into recent events that may be affecting the child’s health. Children with a score above the age-specific cut-offs (ie, a “critical score”) are considered to be at higher risk to suffer from psychological problems. The AQC is an adaptation of the TAS-20 that assesses alexithymia in adults [29]. The AQC consists of 20 items, which are reformulated for children and represent the three factors “Difficulty identifying feelings”, “Difficulty describing feelings” and “Externally oriented thinking”. There are three possible responses to the various items: “not true”, “a bit true”, “true”. The Italian version of the AQC contains four factors, the three of the English version plus a factor related to confused physical perceptions, which focuses on aspects of emotions concerning the body [30]. Higher AQC scores correspond to a greater impairment in the recognition and verbalization of emotions (score > 30.17 defines critical AQC for the age group 7–10 years; score > 28.22 defines critical AQC for the age group 11–14 years) [29, 30]. Critical scores for the CLES-C and ACQ were defined by previous studies in a range of healthy children unaffected by chronic disease [27–30]. The PH-C consists of 18 items that assess somatic arousal, defined as bodily manifestations of autonomic arousal [31]. Elevated levels of somatic arousal (physiological hyperarousal) represent anxiety. In the PH-C, children rate on a 5-point scale (from 1 = never to 5 = all the time) how often they have experienced symptoms such as sweaty hands, feeling of choking, heart pounding, during the past 2 weeks. Higher scores indicate higher levels of physiological hyperarousal.

Disease severity in C1-INH-HAE patients was evaluated using the clinical severity score designed by Bygum and colleagues, based on information collected from patient charts and parent and children interviews [4]. The disease severity score ranges from 0 to 10 and takes into account age at disease onset, clinical manifestations (edema location, occurrence of painful abdominal edema) and treatment experiences (need for long-term prophylaxis). Highest scores indicate more severe disease.

### Statistical analysis

Data were summarized by descriptive analysis. Means and SD were calculated for continuous variables, while absolute values and frequency (percentage) were calculated for categorical variables. Analysis of variance and Chi Square tests were used to examine the significance of the differences between groups for continuous and categorical variables, respectively. Correlations between C1-INH-HAE severity and alexithymia, perceived stress and other parameters were assessed using general linear models or logistic regression analysis when the dependent variable was dichotomous. All tests were two-sided and the level of significance was set at *p* ≤ 0.05 for all analyses.

## Results

Overall, 28 children with C1-INH-HAE, 23 with T1D, and 25 with RA were enrolled in this study (Table 1). Mean age (standard deviation [SD]) in the three groups was 11.8 (3.3), 11.7 (2.9), and 11.1 (2.6) years, respectively. Most patients with C1-INH-HAE (75%) had family members affected by C1-INH-HAE, while a family history was less prevalent in T1D and RA patients. In the C1-INH-HAE group, boys were the majority (67.9%), while in the other two groups, the majority of patients were girls. Frequency of symptoms (swelling and angioedema attacks in C1-INH-HAE patients, hyper- and hypoglycemia in patients with T1D, and joint pain in patients with RA) over the past 12 months was similar among disease groups. Overall, a symptom frequency of ≥2 events per trimester was reported by 48–57% of children over the previous 12 months.

**Table 1:** Baseline demographics

Demographic	Hereditary angioedema	Type 1 diabetes	Rheumatoid arthritis
	*N* = 28	*N* = 23	*N* = 26
Age, yrs., mean (SD)	11.8 (3.3)	11.7 (2.9)	11.1 (2.5)
Gender, *n* (%)			
Female	9 (32.1)	17 (73.9)	16 (61.5)
Male	19 (67.9)	6 (26.1)	10 (38.5)
Family history, *n* (%)			
Yes	21 (75.0)	5 (21.7)	–^a^
No	7 (25.0)	18 (78.3)	24 (100.0)

^a^Family history was unavailable for two patients

SD, standard deviation; yrs., years

On average, children had experienced between 2 and 3 attacks during the previous month (Table 2). With regard to the attacks reported over the past 12 months, most patients had skin (89.3%) and abdominal attacks (82.1%); laryngeal attacks were reported by approximately 40% of patients. The mean (SD) disease severity score was 5.9 (2.1). Disease severity was comparable between male and female patients, with the latter group showing a trend towards a higher mean disease score. In addition, the disease severity score appeared to increase with age, while the number of attacks over the past month showed a decreasing trend with age.

**Table 2:** Clinical characteristics of patients with hereditary angioedema

Clinical characteristic	*N* = 28
Number of attacks during past month, median (range)	2.5 (0–8.0)
Age at disease onset, yrs., mean (SD)	2.4 (0.8)
Use of prophylaxis therapy, *n* (%)	
Yes	4 (14.3)
No	24 (85.7)
Site of angioedema attacks^a^, *n* (%)	
Skin	25 (89.3)
Abdomen	23 (82.1)
Larynx	11 (39.3)
Total disease severity score, mean (SD)	5.9 (2.1)

^a^Referred to attacks experienced during the 12 months preceding the interview

SD, standard deviation; yrs., years

**Fig. 1:**
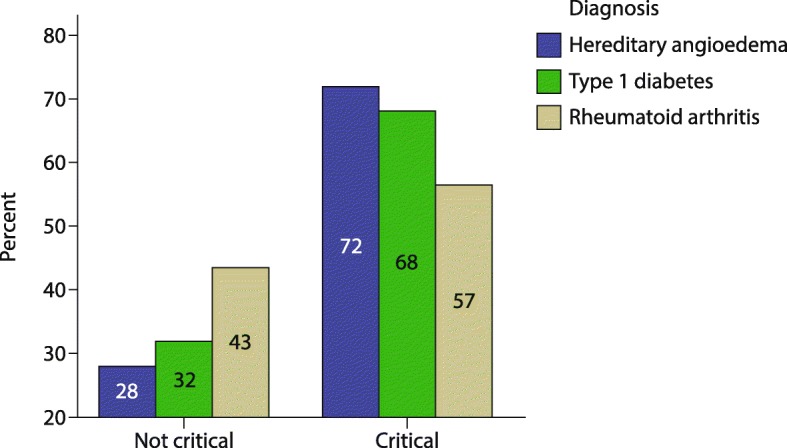
Patients with hereditary angioedema and controls with non-critical and critical alexithymia

According to the results of the analysis of alexithymia using the AQC questionnaire, patients in all three groups showed difficulties in identifying and describing feelings, were prone to externally-oriented thinking, and were moderately confused about their physical perceptions (Table 3). A greater proportion of children with C1-INH-HAE showed a level of alexithymia that was defined as critical, compared with children with T1D or RA (*p* = 0.506; Fig. 1). Of note, C1-INH-HAE children with critical AQC reported a higher mean total disease severity score [6.4 (1.8)] than those with non-critical AQC [5.3 (2.6)].

**Table 3:** Alexithymia in patients with hereditary angioedema and control groups

Disease	DIF	DDF	EOT	CPF	TAS
C1-INH-HAE	11.3 (4.0)	8.9 (2.8)	13.6 (2.7)	5.6 (2.1)	34.4 (6.7)
T1D	13.1 (3.6)	9.9 (3.2)	14.1 (2.3)	6.6 (2.2)	36.5 (6.8)
RA	13.4 (4.4)	9.7 (2.9)	13.5 (2.0)	6.9 (2.5)	36.6 (8.0)

Data are presented as mean (standard deviation). Analysis of variance *p*-values for DIF, DDF, EOT, CPF, and total score were 0.181, 0.452, 0.731, 0.171, 0.480, respectively

*C1-INH-HAE* hereditary angioedema with C1-inhibitor deficiency, *CPF* confusion in physical sensations, *DDF* difficulty describing feelings, *DIF* difficulty identifying feelings, *EOT* externally-oriented thinking, *RA* rheumatoid arthritis, *TAS* total alexithymia score, *T1D* type 1 diabetes

**Fig. 2:**
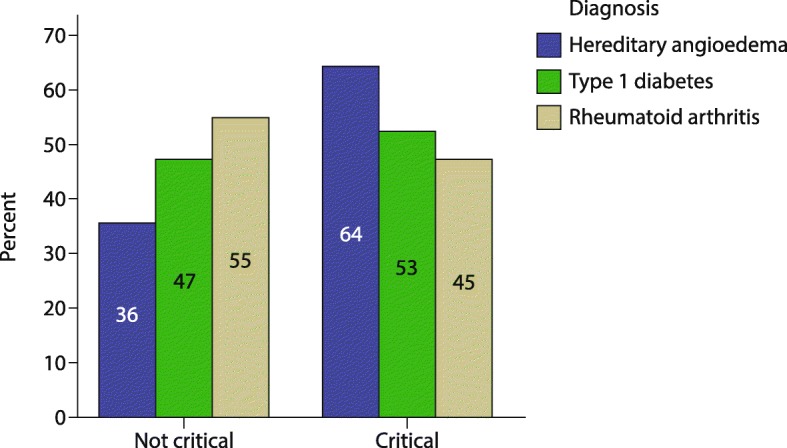
Patients with hereditary angioedema and controls non-critical and critical perceived stress

Compared with children in the two control groups, those with C1-INH-HAE reported more stressful events over the 12 months prior to the study interview. A greater proportion of children in the C1-INH-HAE group (64%) showed critical CLES-C scores compared with children in the T1D and RA groups (53 and 45%, respectively, *p* = 0.401; Fig. 2). As for PH-C scores, children with RA reported the highest mean scores [34.4 (12.5)], followed by children with T1D [30.7 (7.9)] and C1-INH-HAE [29.4 (11.7)]. Differences between groups were not statistically significant (*p* = 0.436).

The analysis of the relationship between C1-INH-HAE severity score and the scores related to the ability to recognize and describe feelings found a statistically significant positive correlation between total severity score and AQC score (*p* = 0.046). No statistically significant correlation was found between the total severity score and CLES-C scores, and between C1-INH-HAE attack frequency and the AQC score. Notably, AQC scores were found to correlate significantly and positively with CLES-C scores (Additional file 1: Table S1). Thus, increasing levels of alexithymia were associated with increasing odds of experiencing critical levels of perceived stress.

## Discussion

The majority of children with C1-INH-HAE in the present study showed critical alexithymia and critical levels of perceived stress, consistent with our previous observations [18]. This was true also for children affected by T1D and RA, suggesting that alexithymia and stress are common features of chronic illnesses in this age group. The proportions of children with critical AQC and CLES-C scores were consistently more elevated in C1-INH-HAE patients compared with the two control groups, indicating that impairment in regulation and processing of emotions as well as stress may be more relevant in C1-INH-HAE children. The severity of C1-INH-HAE was found to correlate significantly and positively with alexithymia, but not with perceived stress. A significant correlation was found also between alexithymia and perceived stress, which suggests that the inability to identify and describe feelings may result in reduced abilities to cope with stress.

Currently we can only speculate about the reasons why children affected by chronic diseases show alexithymia. Several studies have suggested that experiences of adversity early in life, for example poverty, but also psychological distress, can have a negative impact on emotional functioning and other health-related aspects [13, 32, 33]. Thus, the psychological distress associated with the burden of chronic disease during childhood may be a cause of impaired emotional processing and alexithymia. Moreover, in the attempt to avoid triggers, parents may implicitly discourage the contact with emotional states in their children, due to a fear of emotions as triggers of attacks [10]. At the same time, psychological distress may also be a consequence of the high levels of stress perceived by these patients [27].

In our first study investigating psychological factors in children with C1-INH-HAE, all interviewed parents indicated stress and emotions – both positive and negative – as the most frequent triggers of angioedema attacks and, thus, as the factors potentially involved in the high variability of attack occurrence [18]. A recent study investigating various aspects of C1-INH-HAE in Swedish children (median age, 9 years), based on a questionnaire answered mostly by the parents, also found that psychological stress is the most commonly reported trigger of abdominal attacks [34].

The unpredictability of angioedema attacks is often considered as more distressing than the physical symptoms of the attack [3, 6, 10]. The uncertainty associated with angioedema attacks further complicates everyday life, because the planning of any activity can be very difficult [10]. A study evaluating health-related QoL (HRQoL) in children using a validated questionnaire based on child self-report and on parent proxy reports (PedsQL™ 4.0) found that the fear of a sudden angioedema attack causes emotional distress and anxiety [8]. Due to this fear, patients often avoid activities and social life, which further worsens emotional distress and impairment of HRQoL. Other authors have pointed to the psychosocial factors that appear between attacks, including stress driven by uncertainty, as important contributors to the disease burden [14]. As a consequence, interventions that target psychological aspects and help patients to achieve emotional control are increasingly regarded as potential strategies for reducing the chances to trigger an attack [9].

The importance of emotional awareness and the potential of psychological interventions aimed at reducing alexithymia is supported by a number of reports from other therapeutic areas and mostly in adults [35–37]. In a case series including adult patients with chronic musculoskeletal pain, emotion-oriented interventions were associated with clinically relevant improvements in pain intensity, interference, depression and distress, and with a decrease in alexithymia as assessed by the Levels of Emotional Awareness Scale score [35]. The interventions included attributing pain to psychological processes, emotional awareness and expression, and engaging in desired activities despite pain. It was, however, pointed out by the authors that this approach, which attributes pain mainly to mental processes, does not apply to all patients [35]. Indeed, in patients with chronic pain caused by peripheral disease processes or structural alterations, emotional problems are usually the consequence rather than the cause of pain. A study in patients with irritable bowel syndrome (IBS) identified alexithymia as a strong predictor of IBS severity suggesting that altered emotion awareness and processing may result in clinical manifestations of IBS [36, 37]. The authors highlighted that improving the ability to recognize emotions and to deal with them may lead to better treatment outcomes, especially in patients with high alexithymia [37].

Despite a number of limitations, such as the limited sample size and the lack of a control group of healthy children, the present study demonstrates that psychological assessments are feasible in children aged 6–17 years (mean age of the C1-INH-HAE patients, 11.8 years). The comparators in our study were children with other forms of chronic disease, rather than healthy children, but we were also able to show that a high proportion of C1-INH-HAE children have critical scores for alexithymia and perceived stress derived from validation studies conducted in healthy children. Assessment of stress and anxiety in children with chronic diseases are challenging, but the evidence from our studies, as well as from the reports by other authors, shows that children aged ≥7 years are able to evaluate their anxiety [38]. Overall, children continue to be underrepresented in clinical trials and the need for children-specific assessment tools is recognized also in the C1-INH-HAE field. The use of self-reported tools for evaluating anxiety and other psychological factors in children should be encouraged as parent-reported proxy tools may not be always adequate [38].

## Conclusions

In C1-INH-HAE, which is characterized by a very high variability of symptom manifestations, the presence of marked alexithymia might be predictive of even worse outcomes. Our findings suggest that impaired emotional competence may also lead to an increase in perceived stress, which is a possible trigger of angioedema attacks. Perceived stress, emotional competence, and anxiety should be assessed in children with chronic diseases. As recommended by current guidelines for the management of children with C1-INH-HAE, programs for comprehensive patient management should also include psychological interventions, if needed, and encourage the cooperation between physicians and psychologists [7]. Individual and group psychological interventions aimed at improving the ability of children with C1-INH-HAE to recognize their feelings and to cope with them should be offered to children and their parents. Such interventions may help reduce stress levels and, ultimately, control factors that trigger attacks and contribute to the unpredictability of disease manifestations.

## Additional file

Additional file 1: **Table S1.** Binary Logistic Regression analysis: Coddington Life Event Scale (CLES) versus Total Severity Score and Alexithymia Questionnaire for Children (AQC). (DOCX 20 kb)

## Abbreviations

AQC: Alexithymia Questionnaire for Children
C1-INH-HAE: hereditary angioedema with C1-inhibitor deficiency
CLES: Coddington Life Events Scale
CLES-C: Coddington Life Events Scale for children
HRQoL: health-related quality of life
IBS: irritable bowel syndrome
PH-C: Physiological Hyperarousal in children
QoL: quality of life
RA: rheumatoid arthritis
SD: standard deviation
T1D: type 1 diabetes
TAS-20: Toronto Alexithymia Scale 20

## References

[1] Cicardi M, Aberer W, Banerji A, Bas M, Bernstein JA, Bork K (2014). Classification, diagnosis, and approach to treatment for angioedema: consensus report from the hereditary angioedema international working group. Allergy.

[2] Zuraw BL (2008). Clinical practice. Hereditary angioedema. N Engl J Med.

[3] Bygum A, Aygören-Pürsün E, Beusterien K, Hautamaki E, Sisic Z, Wait S (2015). Burden of illness in hereditary angioedema: a conceptual model. Acta Derm Venereol.

[4] Bygum A, Fagerberg CR, Ponard D, Monnier N, Lunardi J, Drouet C (2011). Mutational spectrum and phenotypes in Danish families with hereditary angioedema because of C1 inhibitor deficiency. Allergy.

[5] Caballero T, Aygören-Pürsün E, Bygum A, Beusterien K, Hautamaki E, Sisic Z (2014). The humanistic burden of hereditary angioedema: results from the burden of illness study in Europe. Allergy Asthma Proc.

[6] Lumry WR, Castaldo AJ, Vernon MK, Blaustein MB, Wilson DA, Horn PT (2010). The humanistic burden of hereditary angioedema: impact on health-related quality of life, productivity, and depression. Allergy Asthma Proc..

[7] Farkas H, Martinez-Saguer I, Bork K, Bowen T, Craig T, Frank M (2017). International consensus on the diagnosis and management of pediatric patients with hereditary angioedema with C1 inhibitor deficiency. Allergy.

[8] Engel-Yeger B, Farkas H, Kivity S, Veszeli N, Kőhalmi KV, Kessel A (2017). Health-related quality of life among children with hereditary angioedema. Pediatr Allergy Immunol.

[9] Kessel A, Farkas H, Kivity S, Veszeli N, Kőhalmi KV, Engel-Yeger B (2017). The relationship between anxiety and quality of life in children with hereditary angioedema. Pediatr Allergy Immunol.

[10] Savarese L, Bova M, De Falco R, Guarino MD, Siani G, Valerio P, Freda MF, De Luca Picione R (2017). The role of the meaning-making process in the management of hereditary angioedema. Healthcare and culture: subjectivity in medical contexts. Charlotte, NC: information age publishing.

[11] Caballero T, Maurer M, Longhurst HJ, Aberer W, Bouillet L, Fabien V (2016). Triggers and prodromal symptoms of angioedema attacks in patients with hereditary angioedema. J Investig Allergol Clin Immunol.

[12] Zotter Z, Csuka D, Szabó E, Czaller I, Nébenführer Z, Temesszentandrási G (2014). The influence of trigger factors on hereditary angioedema due to C1-inhibitor deficiency. Orphanet J Rare Dis.

[13] Trudel-Fitzgerald C, Qureshi F, Appleton AA, Kubzansky LD (2017). A healthy mix of emotions: underlying biological pathways linking emotions to physical health. Curr Opin Behav Sci.

[14] Longhurst H, Bygum A (2016). The humanistic, societal, and pharmaco-economic burden of angioedema. Clin Rev Allergy Immunol.

[15] Fouche AS, Saunders EF, Craig T (2014). Depression and anxiety in patients with hereditary angioedema. Ann Allergy Asthma Immunol.

[16] Burns VE, Edwards KM, Ring C, Drayson M, Carroll D (2008). Complement cascade activation after an acute psychological stress task. Psychosom Med.

[17] Wu MA, Casella F, Perego F, Suffritti C, Afifi Afifi N, Tobaldini E (2017). Hereditary angioedema: assessing the hypothesis for underlying autonomic dysfunction. PLoS One.

[18] Freda MF, Savarese L, Bova M, Galante A, De Falco R, De Luca Picione R (2016). Stress and psychological factors in the variable clinical phenotype of hereditary angioedema in children: a pilot study. Pediatr Allergy Immunol Pulmonol.

[19] Salemi M, Di Bella F, Miragliotta A, Perricone R, Guarino MD, Cicardi M (2014). Psychological correlates in subjects with hereditary angioedema (HAE). J Psychol Psychother.

[20] Sifneos PE (1973). The prevalence of 'alexithymic' characteristics in psychosomatic patients. Psychother Psychosom.

[21] Taylor GJ (1984). Alexithymia: concept, measurement, and implications for treatment. Am J Psychiatry.

[22] Taylor GJ, Bagby RM, Parker JD (1991). The alexithymia construct. A potential paradigm for psychosomatic medicine. Psychosomatics.

[23] Kano M, Fukudo S. The alexithymic brain: the neural pathways linking alexithymia to physical disorders. Biopsychosoc Med. 2013;7(1) 10.1186/1751-0759-7-1. 10.1186/1751-0759-7-1 PMC3563604 23302233

[24] Kojima M (2012). Alexithymia as a prognostic risk factor for health problems: a brief review of epidemiological studies. Biopsychosoc Med.

[25] Achenbach TM, Rescorla LA (2000). Manual for the ASEBA preschool forms and profiles.

[26] Achenbach TM, Rescorla LA (2001). Manual for the ASEBA school-age forms and profiles.

[27] Coddington RD (1972). The significance of life events as etiologic factors in the diseases of children. II. A study of a normal population. J Psychosom Res.

[28] Coddington RD, Sogos C, Piperno F, Di Biasi SCLES (2009). Coddington life events scales: manuale. Firenze.

[29] Rieffe C, Oosterveld P, Meerum Terwogt M (2006). An alexithymia questionnaire for children: factorial and concurrent validation results. Pers Ind Diff.

[30] Di Trani M, Tomassetti N, Bonadies M, Capozzi F, De Gennaro L, Presaghi F (2009). An Italian questionnaire for alexithymia for children and adolescents. Psicologia della Salute.

[31] Laurent J, Joiner TE, Catanzaro SJ (2011). Positive affect, negative affect, and physiological hyperarousal among referred and nonreferred youths. Psychol Assess.

[32] Reiss F (2013). Socioeconomic inequalities and mental health problems in children and adolescents: a systematic review. Soc Sci Med.

[33] Winning A, Glymour MM, McCormick MC, Gilsanz P, Kubzansky LD (2016). Childhood psychological distress as a mediator in the relationship between early-life social disadvantage and adult cardiometabolic risk: evidence from the 1958 British birth cohort. Psychosom Med.

[34] Nygren A, Nordenfelt P, Lindfors A, Mallbris L, Björkander J, Wahlgren CF (2016). Swedish children with hereditary angioedema report good overall health and quality of life despite symptoms. Acta Paediatr.

[35] Burger AJ, Lumley MA, Carty JN, Latsch DV, Thakur ER, Hyde-Nolan ME (2016). The effects of a novel psychological attribution and emotional awareness and expression therapy for chronic musculoskeletal pain: a preliminary, uncontrolled trial. J Psychosom Res.

[36] Porcelli P, De Carne M, Leandro G (2014). Alexithymia and gastrointestinal-specific anxiety in moderate to severe irritable bowel syndrome. Compr Psychiatry.

[37] Porcelli P, De Carne M, Leandro G (2017). The role of alexithymia and gastrointestinal-specific anxiety as predictors of treatment outcome in irritable bowel syndrome. Compr Psychiatry.

[38] Nilsson S, Buchholz M, Thunberg G (2012). Assessing children’s anxiety using modified short state-trait anxiety inventory and talking mats: a pilot study. Nurs Res Pract.

