# Supplementary material for: Emotional processes and stress in children affected by hereditary angioedema with C1-inhibitor deficiency: a multicenter, prospective study
Source: Orphanet J Rare Dis. 2018 Jul 13;13:115. doi: 10.1186/s13023-018-0871-x (PMC6043996; doi:10.1186/s13023-018-0871-x)
Supplement: Supplementary file 1 — Table S1. Binary Logistic Regression analysis: Coddington Life Event Scale (CLES) versus Total Severity Score and Alexithymia Questionnaire for Children (AQC). (DOCX 20 kb) [file 13023_2018_871_MOESM1_ESM.docx]

Binary Logistic Regression analysis: Coddington Life Event Scale (CLES) versus Total Severity Score and Alexithymia Questionnaire for Children (AQC)

|  | **B** | **SE** | **Wald** | **df** | **Sig** | **Exp(B)** |
| --- | --- | --- | --- | --- | --- | --- |
| Total Severity Score | –0.298 | 0.251 | 1.408 | 1 | 0.235 | 0.742 |
| AQC Total Score | 0.178 | 0.090 | 3.913 | 1 | 0.048 | 1.195 |
| Familiarity | 0.914 | 1.138 | 0.645 | 1 | 0.422 | 2.494 |
| Constant | –4.182 | 2.725 | 2.356 | 1 | 0.125 | 0.015 |
|  | **Hosmer and Lemeshow Goodness-of-fit Test** | | | | | |
|  | **Chi-square** | | **df** | | **Sig** | |
| Goodness-of-fit Test | 4.702 | | 8 | | 0.789 | |
| Step | **Model Summary** | | | | | |
|  | **–2 Log likelihood** | | **Cox & Snell R^2^** | | **Nagelkerke R^2^** | |
| 1 | 27.177 | | 0.202 | | 0.272 | |

AQC, Alexithymia Questionnaire for Children
